# Latent growth of the non-dilated descending thoracic aorta as a marker of genetic aortopathy

**DOI:** 10.1093/ehjci/jeag056

**Published:** 2026-02-21

**Authors:** Carlos Alberto Campello Jorge, Prabhvir S Marway, Vedika Ramesh, Heather Knauer, Timothy J Baker, Rajani Aatre, Kim Eagle, Marion A Hofmann Bowman, Nicholas S Burris

**Affiliations:** Department of Radiology, University of Michigan, 1500 E. Medical Center Dr, Ann Arbor, MI 48109, USA; Department of Radiology, University of Wisconsin-Madison, 800 University Bay Drive, Suite 210, Rm 224, Madison, WI 53705, USA; Department of Radiology, University of Michigan, 1500 E. Medical Center Dr, Ann Arbor, MI 48109, USA; Department of Radiology, University of Wisconsin-Madison, 800 University Bay Drive, Suite 210, Rm 224, Madison, WI 53705, USA; Department of Internal Medicine—Division of Cardiovascular Medicine, University of Michigan, Ann Arbor, MI, USA; Department of Radiology, University of Michigan, 1500 E. Medical Center Dr, Ann Arbor, MI 48109, USA; Department of Radiology, University of Michigan, 1500 E. Medical Center Dr, Ann Arbor, MI 48109, USA; Department of Internal Medicine—Division of Cardiovascular Medicine, University of Michigan, Ann Arbor, MI, USA; Department of Internal Medicine—Division of Cardiovascular Medicine, University of Michigan, Ann Arbor, MI, USA; Department of Internal Medicine—Division of Cardiovascular Medicine, University of Michigan, Ann Arbor, MI, USA; Department of Radiology, University of Michigan, 1500 E. Medical Center Dr, Ann Arbor, MI 48109, USA; Department of Radiology, University of Wisconsin-Madison, 800 University Bay Drive, Suite 210, Rm 224, Madison, WI 53705, USA

Disease severity in ascending thoracic aortic aneurysm (aTAA) is largely defined by the maximal diameter of the ascending aorta. However, little research has examined the value of quantifying descending aortic involvement in aTAA to better understand disease aetiology and severity. In current practice, if the descending aorta is non-dilated (<3.0 cm), it is considered uninvolved in the aTAA pathology. Yet, in heritable thoracic aortic disease (HTAD), diffuse involvement of the thoracic aorta is common and confers a higher risk of complications, namely type B dissection (TBAD), which can occur at ‘normal’ size.^1,2^

Vascular deformation mapping (VDM) is a validated analysis technique that enables 3D mapping of growth throughout the thoracic aorta using clinical computed tomography angiography (CTA).^3^ Prior studies using VDM have observed growth of non-dilated descending aortic segments in patients with aTAA; however, the significance of this finding remains unclear. The objective of this study was to quantify the frequency of growth in non-dilated descending aorta—termed latent aortic growth (LAG)—in aTAA patients undergoing genetic evaluation to test the hypothesis that LAG is associated with the confirmed diagnosis of HTAD.

We conducted a single-centre, retrospective cohort study (2004–2024) approved by the institutional review board (HUM00133798). Inclusion criteria were (i) diagnosis of ascending aortic dilation; (ii) ≥ 2 electrocardiographically gated CTAs spanning ≥2 years; (iii) native, non-dissected descending thoracic aortic diameter <3.0 cm at baseline; and (iv) evaluation for HTAD, defined as assessment by a medical geneticist or genetic counsellor with clinical phenotyping and/or genetic testing. Patients were stratified based on evaluation results into three mutually exclusive groups: (i) HTAD: Patients with a pathogenic/likely pathogenic (P/LP) variant or a confirmed clinical diagnosis (e.g. meeting Ghent criteria); (ii) variant of uncertain significance (VUS): Patients with inconclusive genetic testing (VUS) and no confirmed clinical diagnosis; and (iii) No confirmed HTAD (Negative): Benign/likely benign variant or negative genetic testing and no clinical criteria for HTAD diagnosis (*Figure 1***, left panel**).

**Figure 1:**
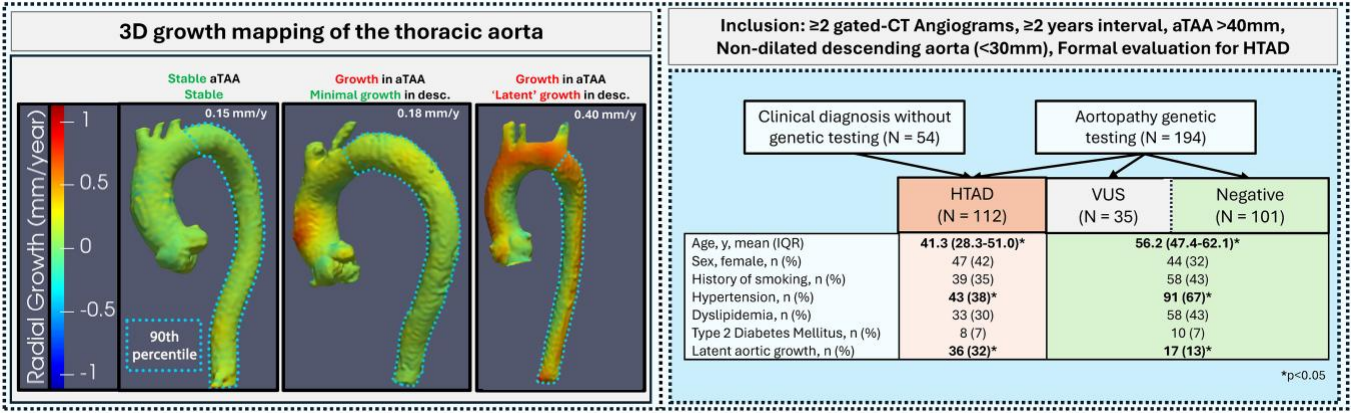
3D growth mapping and study cohort characteristics. Left Panel: Illustration of vascular deformation mapping (VDM) for quantifying aortic growth. This technique generates 3D aortic growth maps from serial CT scans. The colour scale visualizes the rate of radial growth point-by-point in millimetres per year (mm/year), with red showing expansion and green showing stability. From these detailed maps, the 90th percentile growth rate is extracted for the descending thoracic aorta—the specific region of interest outlined by the blue dotted line. The three panels are representative examples demonstrating the visual output of the VDM method across different growth patterns. **Right Panel:** Inclusion flow chart and clinical characteristics. Patients were categorized by clinical and genetic evaluation into HTAD, VUS, or Negative groups. The table compares demographics and risk factors between the HTAD group (Salmon) and the combined VUS/Negative group (green). *P < 0.05 indicating statistical significance.

VDM was used to quantify the 90th percentile growth rate of the descending thoracic aorta over the longest CTA interval. LAG was defined as a descending aortic growth rate >0.3 mm/year. This threshold was selected because it exceeds normal age-related enlargement (≈ 0.1 mm/year), approximates the growth rates of clinically significant ascending aneurysms, and corresponds to the validated sensitivity limits of the VDM technique, which requires an absolute change of ≥0.6 mm over a minimum interval of two years.^3,4^ Statistical comparisons utilized ANOVA, Kruskal–Wallis, and multivariable logistic regression, with *P* < 0.05 considered significant.

The study population comprised 248 patients: 112 (45%) with confirmed HTAD, 37 (15%) with VUS, and 101 (40%) with Negative evaluation (*Figure 1***, right panel**). The HTAD group was younger than the Negative group (median 41.3 vs. 56.9 years; *P* < 0.001) with higher rates of connective tissue disease features (e.g. scoliosis, mitral valve prolapse), aortic root/ascending repair (65.2% vs. 19.8%; *P* < 0.001) and prior DeBakey type II dissection (16.1% vs. 3.0%; *P* < 0.001). Hypertension was less common in the HTAD group compared to Negative (38.4% vs. 64.4%; *P* < 0.001), with lower median systolic blood pressures during surveillance (119.9 vs. 125.6 mmHg; *P* = 0.001).

Overall, 21.4% (53/248) of the cohort demonstrated LAG. When stratified by group, LAG was detected in 32.1% of HTAD, 22.9% of VUS, and 8.9% of Negative patients (*P* < 0.001). LAG presence was associated with greater odds of confirmed HTAD vs. a Negative result [OR 4.8 (95%CI: 2.2–10.7), *P* < 0.001]. LAG frequency was higher in the VUS compared to Negative group (22.9% vs. 8.9%, *P* = 0.041). Median (IQR) descending growth rates were: HTAD 0.21 (0.14–0.39) mm/year, VUS 0.21 (0.14–0.30) mm/year, and Negative 0.17 (0.11–0.22) mm/year (HTAD vs. Negative *P* < 0.001, VUS vs. Negative *P* = 0.026).

By multivariable logistic regression, LAG was independently associated with increased odds of HTAD diagnosis [OR 4.69 (95%CI: 1.63–13.53), *P* = 0.004]. Conversely, age [OR 0.93 (95% CI: 0.91–0.96), *P* < 0.001] and presence of BAV [OR 0.31 (95% CI: 0.12–0.81), *P* = 0.016] were associated with lower odds of HTAD diagnosis. Hypertension, hyperlipidaemia, sex and smoking status were not significant predictors of HTAD diagnosis. Results were unchanged when VUS and Negative groups were combined for analysis.

The key finding of this study is that LAG, a previously unexamined phenomenon, is detectable by VDM in normal-appearing descending aortic segments and may represent a marker of a more diffuse and potentially more severe disease phenotype. The frequency of LAG increased in parallel with HTAD probability, being highest in confirmed HTAD, intermediate in VUS, and lowest in Negative cases.

The association between genetic aortopathy and LAG is mechanistically plausible given that genetic mutations affect all aortic tissue despite differing degrees of phenotypic dilation due to segment-specific wall composition, haemodynamics, and embryological origins.

Our findings could be particularly relevant for the management of patients with VUS results. This subgroup often presents a clinical challenge due to uncertain pathogenicity. if the association of LAG with confirmed HTAD diagnosis is validated in larger, external cohorts, this imaging phenotype may be useful in helping clarify the clinical significance of VUS results.

Despite limitations including its retrospective design, requitement for serial imaging and inability to adjust for all pharmacologic treatments, this study suggests that LAG is a quantitative imaging biomarker of diffuse aortopathy that may improve understanding of genotype–phenotype correlations and refine patient-specific risk stratification beyond maximal aortic diameter, warranting validation in larger, prospective cohorts.

## Data Availability

The derived data that support the findings of this study are available from the corresponding author upon reasonable request. The raw imaging data are not available to share due to restrictions regarding patient privacy and institutional review board approval. The code underlying the vascular deformation mapping technique is proprietary intellectual property and is not available for public distribution.

## Data access and responsibility

N.S.B. had full access to all the data in the study and takes responsibility for the integrity of the data and the accuracy of the data analysis.
